# Comparative evaluation of the Ifakara tent trap-B, the standardized resting boxes and the human landing catch for sampling malaria vectors and other mosquitoes in urban Dar es Salaam, Tanzania

**DOI:** 10.1186/1475-2875-8-197

**Published:** 2009-08-12

**Authors:** Maggy Sikulu, Nicodem J Govella, Sheila B Ogoma, John Mpangile, Said H Kambi, Khadija Kannady, Prosper C Chaki, Wolfgang R Mukabana, Gerry F Killeen

**Affiliations:** 1 Department of Zoology, University of Nairobi, PO Box 30197-00100 GPO Nairobi, Kenya; 2 Ifakara Health Institute, Coordination Office, PO Box 78373, Kiko Avenue, Mikocheni A, Dar es Salaam, United Republic of Tanzania; 3 Durham University, School of Biological and Biomedical Sciences, South Road, Durham, DH1 3LE, UK; 4 Dar es Salaam City Council, Ministry of Regional Administration and Local Government, United Republic of Tanzania; 5 Liverpool School of Tropical Medicine, Vector Group, Pembroke Place, Liverpool, L3 5QA, UK

## Abstract

**Background:**

Frequent, sensitive and accurate sampling of *Anopheles *mosquitoes is a prerequisite for effective management of malaria vector control programmes. The most reliable existing means to measure mosquito density is the human landing catch (HLC). However, the HLC technique raises major ethical concerns because of the necessity to expose humans to vectors of malaria and a variety of other pathogens. Furthermore, it is a very arduous undertaking that requires intense supervision, which is severely limiting in terms of affordability and sustainability.

**Methods:**

A community-based, mosquito sampling protocol, using the Ifakara tent trap-B (ITT-B) and standardized resting boxes (SRB), was developed and evaluated in terms of the number and sample composition of mosquitoes caught by each, compared to rigorously controlled HLC. Mosquitoes were collected once and three times every week by the HLC and the alternative methods, respectively, in the same time and location.

**Results:**

Overall, the three traps caught 44,848 mosquitoes. The ITT-B, HLC and SRB caught 168, 143 and 46 *Anopheles gambiae s.l*. as well as 26,315, 13,258 and 4,791 *Culex *species respectively. The ITT-B was three- and five-times cheaper than the HLC per mosquito caught for *An. gambiae *and *Cx*. Species, respectively. Significant correlations between the numbers caught by HLC and ITT-B were observed for both *An. gambiae s.l*. (P < 0.001) and *Cx*. species (P = 0.003). Correlation between the catches with HLC and SRB were observed for *Cx*. species (P < 0.001) but not *An. gambiae s.l*. (P = 0.195), presumably because of the low density of the latter. Neither ITT-B nor SRB exhibited any obvious density dependence for sampling the two species.

**Conclusion:**

SRBs exhibited poor sensitivity for both mosquito taxa and are not recommended in this setting. However, this protocol is affordable and effective for routine use of the ITT-B under programmatic conditions. Nevertheless, it is recommended that the trap and the protocol be evaluated further at full programmatic scales to establish effectiveness under fully representative conditions of routine practice.

## Background

Monitoring and evaluation of malaria control interventions and their associated impact on malaria burden is essential for understanding progress, successes and challenges in any malaria control effort [1]. In order to accurately estimate and manage the burden of a disease and measure the trends in malaria transmission intensity, more practical and cost-effective survey instruments and methods are needed to monitor the densities of the adult mosquito populations [2,3].

In urban Dar es Salaam, the Urban Malaria Control Programme (UMCP) relies upon the human landing catch (HLC) for entomological surveys of malaria vectors, transmission intensity and for evaluation of regular larvicide application [4-6]. Nonetheless, the HLC is difficult to supervise, unreliable, expensive, labour-intensive and requires skilled catchers. It is also not representative of true human exposure as it is usually implemented by adult males who remain awake and seated all night. However, the most serious problem arises when human participants are at an increased risk of malaria infection [7]. Many other methods e.g. the CDC light trap and the Mbita bed net trap have been employed and evaluated in urban Dar es Salaam as alternatives to HLC but none has proven to approach adequate sensitivity [8].

**Figure 1:**
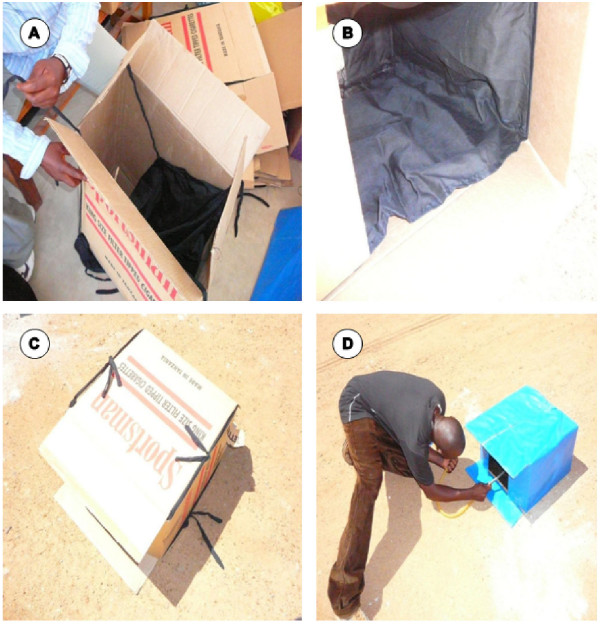
**Photographs of the standardized resting boxes (SRB) used in this study**. Panels A and B illustrate how the boxes are made, panel C demonstrates the way to install them, and panel D demonstrates how to recover resting mosquitoes.

Elsewhere, resting boxes have been used to sample mosquitoes, relying on the widely observed phenomenon that they congregate in diurnal resting places which are dark and cool [9]. Boxes are generally placed on the ground with the opening facing west to minimize the influence of direct sunlight during the early part of the day. In well-shaded areas, the exact direction of the open end becomes less important [2,7,9]. It has been shown that female mosquitoes generally prefer larger and natural resting sites over smaller and artificial resting sites, respectively [10] and that in most cases the numbers of mosquitoes collected do not correlate with the results of host-seeking collections baited with humans [11]. This study investigated the ability of these boxes as outdoor devices for sampling host-seeking mosquitoes in urban Dar es Salaam where they had never been assessed before (Figure 1).

A new device for outdoor sampling adult mosquitoes called the Ifakara tent trap-B (ITT-B) has been developed and evaluated in both rural and urban Tanzania [8]. This trap operates passively all night long without skilled personnel and is designed to prevent exposure of the human volunteer that acts as bait by sleeping inside it. Field studies, in both rural Kilombero Valley and urban Dar es Salaam, have established that the new trap is efficacious in the sense that it has sufficient sensitivity to represent a viable alternative to HLC [8]. This study evaluated the effectiveness of the ITT-B and SRB relative to that of the HLC under field conditions of the Dar es Salaam's UMCP.

## Methods

### Study site

The study was carried out in Dares Salaam where a new Urban Malaria Control Programme has recently been re-initiated and a variety of associated studies on malaria vectors have been carried out [4-6,12-15]. The area experiences modest malaria transmission intensity with an entomologic inoculation rate (EIR) [16,17] of approximately one infectious bite per person per year [4,6]. The main malaria vectors are members of the *An. gambiae *complex that predominantly feed out-doors [5].

Dar es Salaam is a coastal city in Tanzania (6'46' S Latitude and 39'14 E Longitude) with approximately 2.7 million inhabitants living in an administrative region which covers a total area of 1400 km^2 ^[18]. Dar es Salaam has two rainy seasons: the short rains from late October to early December and the long rains from March to June. The climate is warm and tropical, with temperatures averaging 27°C (80°F) and rainfall varying from 750 to 1,400 mm per year. During the dry season temperatures often exceed 35°C.

The UMCP covers 15 wards of urban Dar es Salaam, encompassing a surface area of 55 km^2 ^with a total population of 609,514 people [4]. It operates primarily at the grassroots level through street health committees, using a community-based system originally developed by one of the three municipal councils (Ilala) that comprise the administrative region of Dar es Salaam. Presently, the program operates in five wards in each of the three municipalities (Ilala, Kinondoni and Temeke) as a community-based pilot-scale program [14]. In 2004, the UMCP recruited and provided preliminary training to teams of Community Owned Resource Persons (CORPs) who performed weekly surveys of mosquito breeding habitats [12]. Operational larviciding in three selected wards with *Bacillus thuringiensis *var *israelensis *commenced in 2006 [4,6]. Currently, the UMCP implements four major activities in all the three municipalities: namely larval control, larval surveillance, adult mosquito surveillance and household parasitological surveys [4-6,14]. This study was based within the sampling frame and reporting system of the routine adult mosquito surveillance programme, which conducts monthly sampling of mosquitoes by HLC at 268 location distributed across the 15 wards of the UMCP [5,6].

### Experimental design and selection of the sampling site for the three traps

The study was carried out in 12 wards in the study area of the Urban Malaria Control Program. One neighbourhood (*mtaa *in Kiswahili) in each ward was randomly selected for this study. In each selected neighbourhood, four HLC sites already existed in four Ten Cell Units (TCUs) which were deliberately chosen to be well distributed across the neighbourhood and as close to potential breeding sites as possible. For each pre-existing HLC site, a nearby (100–300 meters away) house was selected arbitrarily for both application of the ITT-B and the SRB. Therefore, in each neighbourhood, eight houses from different TCUs were used for the three sampling methods: four houses for the HLC and four houses for the ITT-B and the SRB, totaling 48 houses for the HLC and 48 for the combined ITT-B and SRB methods, respectively. Concomitant sampling with ITT-B and SRB began in the first enrolled wards in December 2007 and the last of the 12 wards had begun by March 2008. Data and mosquitoes collected up to end of June 2008 were included in this analysis, spanning a period of between seven and four months for each of the 12 wards.

### Field mosquito collection and processing

Routine human landing catch was conducted outdoors once a week in each neighbourhood by one catcher working from 18.00 to 06.00 hours for a period of 45 minutes every hour, allowing the catcher to have a 15 minutes break. To minimize the possibility of data fabrication by the catchers, they were obliged to record the approximate number of each relevant mosquito taxon in their catches for each hour as they finished them. Moreover, spot checks were conducted inconsistently, unpredictably and at arbitrary times of the night by a team of 4 supervisors. The mosquitoes caught were collected by the project vehicle the following morning and taken to the laboratory for further processing.

A protocol for sampling malaria vectors and other mosquitoes using the ITT-B and SRB was developed to enable community members to trap, record and submit samples of malaria vectors without any night-time supervision and only occasional contact with program staff. This protocol was used to evaluate the sensitivity of the ITT-B and the SRB relative to that of carefully controlled HLC as follows: Prior to the supply of materials for ITT-B and SRB experiments, demonstrations were provided to train the community-based staff on correct use of the two traps. The operators were supplied with all the necessary materials that allowed them to continuously collect mosquitoes for a period of one week while recording them on a form they were provided with. Mosquitoes trapped in the ITT-B were carefully aspirated using hand-held aspirators and placed into paper cups, once in the middle of each night (00.00–01.00) and then early in the morning the next day (05.00–06.00). Operators were allowed to choose, at their own discretion, which nights of every week they slept in the traps and what time they entered and left the trap, under the condition that they recorded these dates and times. While still in paper cups, the mosquitoes were suffocated with a small ball of cotton wool soaked in petroleum ether. The dead mosquitoes were then transferred into smaller silica gel-filled containers for storage and preservation with a label indicating the ward, *mtaa*, site and day of collection.

Resting boxes were installed nearby (10–20 m) the ITT-B in each neighbourhood. The boxes were emptied between 06.00 and 08.00 in the morning of each working day using hand-held aspirators. Since experiments with ITT-B and SRB ran concurrently, suffocation, preservation and submission to the laboratory was accomplished in exactly the same way and at the same time as those from the ITT-B.

### Laboratory processing and analysis

All the mosquitoes collected in the field by HLC were taken to the laboratory and killed by suffocation with chloroform. For mosquitoes caught by ITT-B and SRB, this process was completed in the field by the trap operators who submitted their samples for identification and laboratory processing after a one week period of sampling. In the laboratory, all mosquitoes were identified morphologically using taxonomic keys [19] according to sex as males or females, morphologically as *An. gambiae s.l., Anopheles funestus, Anopheles coustani, Cx*. species, or *Aedes *species while the abdominal status was scored as gravid/semi-gravid, fed or unfed for all the *An. gambiae s.l*. and for a manageable proportion of *Cx*. species. All *An. gambiae s.l*. caught by the three trapping methods were subsequently desiccated over silica gel and kept at room temperature until they were further processed.

A wing or a leg of every *An. gambiae s.l*. mosquito caught was analyzed by PCR to identify its exact species within the *An. gambiae *complex [20]. An enzyme-linked immunosorbent assay (ELISA) using a monoclonalantibody that recognizes a repetitive epitope on the circumsporozoiteprotein of *Plasmodium falciparum *was used to assessmalaria sporozoite infection status in each individual *An. gambiae s.l*. [21].

### Data handling and analysis

All data handling and analysis was conducted with Microsoft Excel^® ^2007 and SPSS^® ^15.0. The only mosquito taxa considered for analysis were *An. gambiae s.l*. and *Cx*. species because these were the only ones for which sufficient numbers were collected throughout the study period.

To allow direct comparison with HLC conducted in the same area and in the same week, data was first aggregated by station and week, giving a total of 48 mean catches for matching station-week combinations over a period of 30 weeks. Prior to this analysis step, the numbers in each catch (x) were normalized by transforming to log_10 _[x+1] [3]. The relationship between catches by ITT-B or SRB and that of the HLC, in the same week and station, was initially assessed using simple Pearson's linear correlation method. Regression using generalized estimating equations was used to test for density dependence of the relative sampling efficiency of the ITT-B and SRB methods relative to the sum of the ITT-B and the HLC. On several occasions, the three traps recorded zero values for *An. gambiae s.l*. mosquitoes even after aggregation by station-week so no logical comparison could be made and these data were discarded. Since divisions by zero gives infinite values, data for several week-site observations were sorted by the sum of the catches for the traps (alternative plus the reference) and then aggregated by this sum with the mean of each of the two catches as the summary variables. The mean catch of the alternative collection methods divided by the mean catch of the reference method was treated as the dependent variable with a log link function and a gamma distribution for *An. gambiae s.l*. and a normal distribution for *Cx*. species. The sum of the alternative and the reference methods was treated as a continuous independent variable in the model.

To test for consistent variations in species composition, sporozoite prevalence and abdominal condition of the mosquitoes sampled by the different traps, binary logistic regression in SPSS was used. Each outcome was treated as a binary variable with trap design as an independent categorical factor in the model. The results of abdominal status and sibling species identity were expressed as binary outcomes: fed (partially or fully-fed) versus non-fed (gravid or unfed) and *An. gambiae s.s*. versus *An. arabiensis*, respectively, as described previously [8]. Although sporozoite infection status was determined in the laboratory and the dependence of sporozoite prevalence upon trap type was tested for using a similar statistical approach, the number of mosquitoes caught was not sufficient to enable meaningful conclusions to be reached regarding this relatively rare fraction of the vector population.

### Ethical consideration and informed consent

Informed consent was obtained from all the participants, namely the household owners and the mosquito catchers. Moreover, thick and thin blood smears were taken from all the participants whenever they complained of fever to examine the presence of malaria parasites. When found positive, they were treated with Coartem^® ^(artemether-lumefantrine).

## Results

### Overall performance of the three trapping method

A total of 44,848 mosquitoes were collected during the entire study period of seven months. The composition of the sample was 98.9% *Cx*. species, 0.8% *An. gambiae s.l*., 0.2% *Aedes *species and 0.1% *An. coustani*. The ITT-B, HLC and SRB accounted for 59%, 30% and 11% of the total number of mosquitoes caught respectively. Over the entire sampling period, the SRB caught only 46 and 4,791 *An. gambiae s.l*. and *Cx*. species, respectively. The total catches of *Cx*. species and *An. gambiae s.l*. are outlined in further detail in Table S1 (Additional file 1).

**Figure 2:**
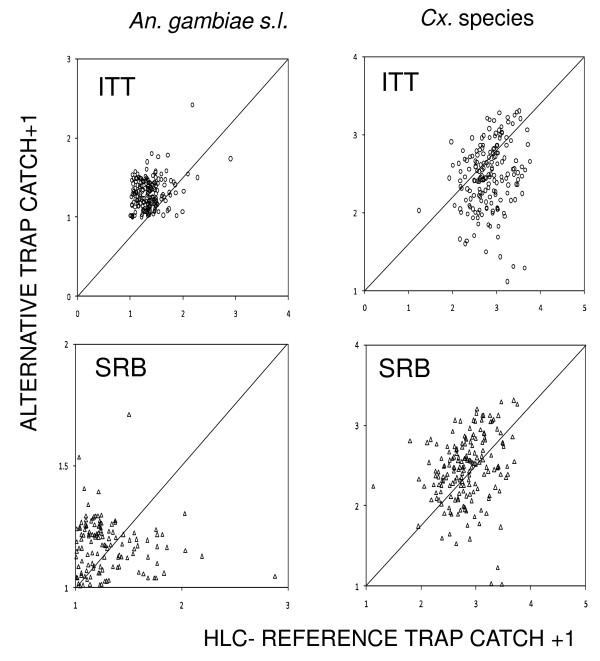
**Relationship between the log of the mean weekly numbers of female *An. gambiae s.l*. and *Cx*. species**. It includes on overall 48 sampling stations over a period of 30 weeks. All values (*X *or *Y*) are presented as *X + 1 + S *or *Y + 1 + S *where S is a random number between 0 and 0.3 added to allow separation and visualization of otherwise identical data points.

**Figure 3:**
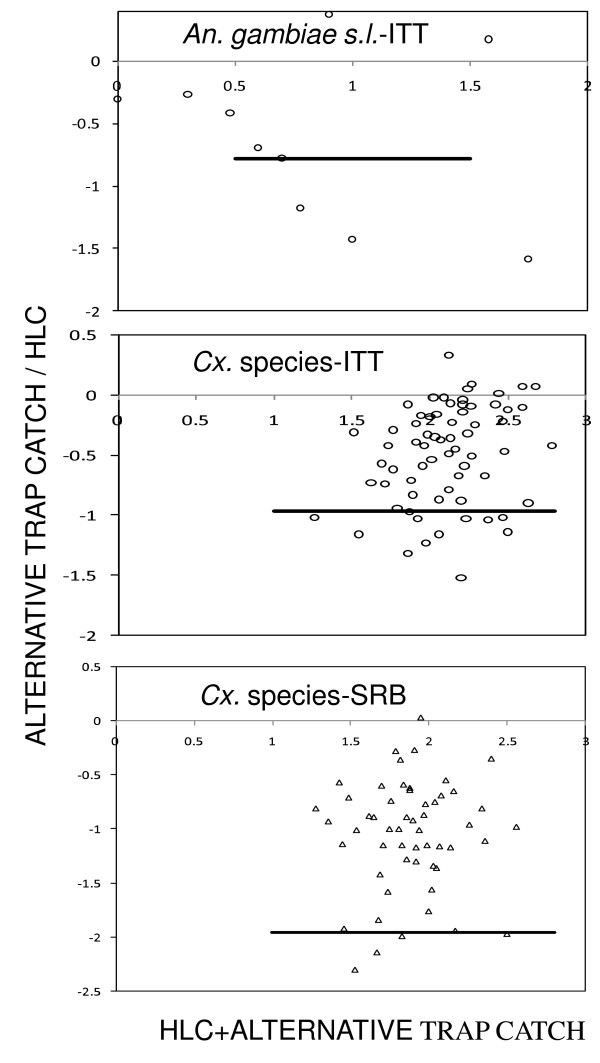
**Density dependence for the relative sampling efficiency of the ITT-B, HLC and SRB for *An. gambiae s.l *and *Cx*. species**. Each point on X axis show the sum number of female *An. gambiae s.l. and Cx*. species caught by the HLC and the alternative trap with several week-site observations. Solid lines depict the density-dependent sampling efficiency model.

There was a significant correlation between the mean weekly numbers of female *An. gambiae s.l*. caught by the ITT-B and the HLC for both *Cx*. species and *An. gambiae s.l*. in the 48 sampling sites (Table S2 (Additional file 2) and Figure 2). However, there was no correlation between the SRB and the HLC for *An. gambiae s.l*. even though a significant correlation existed for the *Cx*. species. Both the ITT-B and SRB showed no density dependence for the relative sampling efficiency of *An. gambiae s.l*. and *Cx*. species (Table S3 (Additional file 3) and Figure 3).

### Dependence of abdominal condition and species composition upon trapping method

The distribution of abdominal conditions observed for *Cx*. species and *An. gambiae s.l*. is presented in Table S4 (Additional file 4). An abdominal condition was determined for 12,776 *Cx*. species and 305 *An. gambiae s.l *No difference was observed between proportions of fed *An. gambiae s.l*. captured by the ITT-B and the HLC. Furthermore, for *Cx*. species, the ITT-B sampled a significantly higher number of fed mosquitoes than the HLC.

Of the 268 female *An. gambiae s.l*. analyzed by PCR for sibling species identification, an amplification success rate of 67.7% was obtained, leaving 87 specimens undetermined (Table S5) (Additional file 5). Of the successful amplifications, 83.3% were *An. gambiae s.s*, 15.5% *Anopheles arabiensis *with the remaining 1.2% being *Anopheles merus*. Both *An. gambiae s.s. and An. arabiensis *were caught by all three sampling methods but notably the SRB yielded comparatively fewer *An. arabiensis *while only the HLC recorded *An. merus *(two specimens). Analysis using binary logistic regression showed no significant difference in proportion of *An. gambiae s.s*. versus *An. arabiensis *sampled by the three traps.

The sporozoite prevalence did not vary significantly between the mosquitoes sampled by the three trapping methods simply because the sample size was too small to make a meaningful analysis.

### Evaluation of the protocol in terms of cost-effectiveness

Table S6 (additional file 6) summarizes the initial cost per week per sampling station, running costs per week per sampling station, total cost and the cost of sampling one mosquito caught for both the ITT-B and the HLC. In all cases, the HLC was more costly than the ITT-B. For example, it was found out that weekly sampling with the HLC (one night of collection) in one sampling station was roughly equivalent in cost to three weeks of sampling with the ITT-B (nine nights of collection) distributed across 3 sampling stations. Also, ITT-B was three and five times cheaper than the HLC per mosquito caught for *An. gambiae s.l*. and *Cx*. species, respectively.

## Discussion and conclusion

More *An. gambiae s.l*. were caught by the ITT-B than by the HLC or SRB. The ITT-B and SRB caught between 35% and 15%, respectively, of the number of *An. gambiae s.l*. caught per night by HLC. Note, however, that by applying the ITT-B for three nights in the same sampling site, its relative sensitivity per week matched the HLC for *An. gambiae s.l*. and exceeded it for *Cx*. species. Interestingly, male mosquitoes of almost all the species sampled were found more frequently in the ITT-B than any other sampling method. It should be noted that male mosquitoes are just as useful an indicator of success or failure of a larval control programme even though they do not cause disease. Furthermore, male mosquitoes play an essential role in the life cycle of all mosquitoes and monitoring systems for genetic control strategies such as the release of sterile or genetically modified mosquitoes. The results of this study show that the ITT-B was the most efficient method for collecting *An. gambiae s.l*. and *Cx*. species per week because it was possible to conduct more intensive sampling with far less effort. SRB, normally considered as the method of choice for recovering resting mosquito populations in a variety of ecological settings, was insufficiently sensitive in urban Dar es Salaam.

Overall, the ITT-B was by far the most cost-effective sampling method. On the basis that the trap can handle more sampling nights than the HLC at a dramatically reduced cost, more sensitive, extensive, intensive and representative measurements of biting density can be determined over larger sampling areas. The fact that the ITT-B samples the vectors with minimal supervision while the HLC requires intense scrutiny and correspondingly substantial resources, are the primary reason for the difference in their overall costs. Whereas HLC involved daily use of a vehicle to distribute the sampling materials to the respective sampling sites, spot checks as well as picking the vectors the next day, the ITT-B involved none of these. The other major differential cost associated with the HLC was the diagnosis and treatment of the HLC catchers in case of any reported fever. By comparison, the ITT-B requires little or no maintenance so after the initial, expensive outlay of purchasing the traps themselves, it is remarkably affordable because these are very durable and the procedure does not require skillful personnel, intensive supervision or medical expense.

The failure of the ITT-B to reduce the proportion of blood fed mosquitoes suggests that exposure of the occupants does in fact occur, probably during the collection process which necessitates opening of the long zipper that bisects the protective panel. Subsequent follow up discussions with the operators revealed that indeed they do receive bites during the collection process. Therefore, an improved design will be required for routine use. One major disadvantage of the ITT-B often reported by the catchers was the fact that the trap was too heavy to be moved from one sampling station to the next by a single person. This problem was later solved by supplying the operators with bicycles. Also, occasionally the trap was reported to attract other insects but none of these were confirmed to be potential mosquito predators. Otherwise, the protocol was generally well accepted by the trap operators and appears to be easy enough for performance to be maintained with relatively modest incentives.

On the other hand, the SRB proved to be very impractical and on several occasions they were either soaked by rain or stolen. It also often proved difficult to retrieve the mosquitoes from the SRB. These problems, combined with their poor sensitivity and other sundry logistical matters appear to rule out the SRB as a candidate tool for routine mosquito sampling in the city of Dar es Salaam. The SRB have been evaluated previously in terms of efficacy and found to correlate poorly with the HLC [11] and this study reinforces that view. In earlier studies, it was found out that the proportion of adult mosquitoes resting in man-made shelters depended on the availability of alternative resting sites which varies according to location and changes seasonally [7] and in a recent study that female mosquitoes prefer larger resting sites over smaller ones [10]. Combining these considerations with the poor sensitivity observed, it appears that SRB are unlikely to provide a useful alternative to the HLC for sampling host-seeking malaria vectors in urban Dar es Salaam, particularly under operational conditions.

The correlation results obtained for ITT-B from this effectiveness trial were slightly different from those of efficacy trials by others [3,8,22,23] (Table S2) (Additional file 2). For example, previous efficacy trials by Govella and others [8] recorded a much stronger correlation between ITT-B and HLC, than seen in this study. This is most probably because this study was carried out under conditions that involved minimal supervision compared to the intensely controlled efficacy trials. Another likely contributor to this weakened association is the fact that more sampling stations across a very heterogeneous environment were included in this study compared to the relatively few sampling stations for the efficacy trials. Nonetheless, the significant positive correlation between the HLC and the ITT-B (Additional file 2) suggest that this approach may be very useful in programmatic setting and provides a reasonably sensitive and accurate reflection of true mosquito biting densities.

Although the use of window traps installed in existing houses and emptied by resident community-based workers has been described as an effective tool for routine monitoring of indoor-residual spray programmes in southern Africa [24], no other effectiveness study of this kind has been reported for malaria vector trapping methods. The ITT-B not only represents an option for more accurate and representative measurement of human biting rate over a large sampling area, it is also practical and affordable to use in community-based sampling schemes. Nevertheless, it is recommended that the trap be evaluated in the longer term and on full programmatic scales until the effectiveness of this approach in fully representative conditions of routine practice is established. However, the largest remaining concern is probably the surprisingly high proportion of blood-fed mosquitoes caught, suggesting the design needs to be adapted to avoid human exposure during the empting process before it can be adopted as a routine mosquito-trapping tool.

## Competing interests

The authors declare that they have no competing interests.

## Authors' contributions

MS developed the protocol for mosquito sampling, supervised the field work, recorded and analyzed the data and drafted the manuscript. NJG designed the Ifakara tent trap-B design and assisted in interpretation of the results. JM, SHK and PC supervised the field work and facilitated the implementation of the protocol. SBO assisted in data collection, data recording and implementation of the protocol. KK was involved in the planning and implementation of the protocol. WRM helped in drafting the study objectives and the manuscript. GFK conceived the study and oversaw the development of the experimental design, interpretation of the results, data analysis and drafting of the manuscript. All authors read and approved the final manuscript.

## Supplementary Material

Additional file 1 **Summary of the totals, means and relative sensitivity *of An. gambiae s.l*. and *Cx*. species caught by the ITT-B, HLC and SRB**. Summary of *An. gambiae s.l*. and *Cx*. species catches by the three traps. Click here for file

Additional file 2 **Results from this study compared to other studies evaluating correlation between the HLC catches and alternative traps for female *An. gambiae s.l***. The data compares correlation between the catches of *An. gambiae s.l*. caught by the HLC and the alternative traps in this study and previous efficacy studies. Click here for file

Additional file 3 **Regression analysis using generalized estimating equations (GEE) to determine density dependence relative sampling efficiency of the ITT-B and the SRB for *An. gambiae s.l*. and the *Cx*. species**. Statistical analysis to indicate the sampling efficiency of the ITT-B and the HLC in terms of the vector density. Click here for file

Additional file 4 **Abdominal condition scored by the three traps for *An. gambiae s.l*. and *Cx*. species and the influence of each trap on the fed mosquitoes determined by binary logistic regression**. The data represent statistical analysis of the abdominal status of the three traps. Click here for file

Additional file 5 **Species composition of *An. gambiae *complex for the ITT-B, HLC and SRB and the influence of each trap upon the proportion of *An. gambiae s.s*. sampled, as determined by binary logistic regression**. The data presented is a summary of statistical analysis of the species composition of the three traps as determined by PCR. Click here for file

Additional file 6 **Comparative evaluation of cost effectiveness of the ITT-B and the HLC for weekly sampling and sampling a single *An. gambiae s.l*. and *Cx*. species**. A summary of the cost of using the ITT-B and the HLC for sampling *An. gambiae s.l*. and *Cx*. species. Click here for file
