# Supplementary material for: Comparative evaluation of the Ifakara tent trap-B, the standardized resting boxes and the human landing catch for sampling malaria vectors and other mosquitoes in urban Dar es Salaam, Tanzania
Source: Malar J. 2009 Aug 12;8:197. doi: 10.1186/1475-2875-8-197 (PMC2734863; doi:10.1186/1475-2875-8-197)
Supplement: Additional file 1 — Summary of the totals, means and relative sensitivity of An. gambiae s.l. and Cx. species caught by the ITT-B, HLC and SRB. Summary of An. gambiae s.l. and Cx. species catches by the three traps. [file 1475-2875-8-197-S1.pdf]

Table S1: A summary of the totals, means and relative sensitivity *of An. gambiae s.l.* and *Cx.* species caught by the ITT-B, HLC and SRB

Collection method	Trap nights	*An. gambiae s.l.*					*Cx. species*				
		Total catch			Mean catch[a]	Relative sensitivity[b]	Total catch			Mean catch[a]	Relative sensitivity[b]
		Male	Female	Total			Male	Female	Total		
ITT-B	606	33	135	168	0.27	0.35	8634	17689	26315	43.42	0.63
SRB	379	19	27	46	0.12	0.15	1786	3005	4791	12.64	0.18
HLC	195	0	143	143	0.78	1.00[c]	279	12979	13258	67.99	1.00[c]

[a]=Mean *An. gambiae s.l.* and *Cx. species* caught per night.

[b]=Crude estimate relative to HLC, calculated by dividing the mean trap catch by that of the HLC.

[c]=Reference trap.
