# Supplementary material for: Comparative evaluation of the Ifakara tent trap-B, the standardized resting boxes and the human landing catch for sampling malaria vectors and other mosquitoes in urban Dar es Salaam, Tanzania
Source: Malar J. 2009 Aug 12;8:197. doi: 10.1186/1475-2875-8-197 (PMC2734863; doi:10.1186/1475-2875-8-197)
Supplement: Additional file 2 — Results from this study compared to other studies evaluating correlation between the HLC catches and alternative traps for female An. gambiae s.l. The data compares correlation between the catches of An. gambiae s.l. caught by the HLC and the alternative traps in this study and previous efficacy studies. [file 1475-2875-8-197-S2.pdf]

Table S2: Results from this study compared to other studies evaluating correlation between the HLC catches and alternative traps for sampling female *An. gambiae s.l.* and *Cx.* species

		An. gambiae s.l.		*Cx.* species	
Source	Alternative collection method	Versus HLC reference method			
This study		r^2	P	r^2	P
	ITT-B	0.104	<0.001	0.049	0.003
	SRB	0.115	0.195	0.167	<0.001
Other studies					
Govella *et al.*, 2009 [8]	ITT-B	0.731	<0.001	NA	NA
Mbogo *et al.*, 1993 [23]	CDC-light trap	0.409	<0.001	NA	NA
Magbity *et al.*, 2002 [22]	CDC- light trap	0.521	<0.001	NA	NA
Lines *et al.*, 1991 [3]	CDC- light trap	0.723	<0.001	NA	NA

CDC= Centre’s for disease control

NA=Not applicable
