# Supplementary material for: Comparative evaluation of the Ifakara tent trap-B, the standardized resting boxes and the human landing catch for sampling malaria vectors and other mosquitoes in urban Dar es Salaam, Tanzania
Source: Malar J. 2009 Aug 12;8:197. doi: 10.1186/1475-2875-8-197 (PMC2734863; doi:10.1186/1475-2875-8-197)
Supplement: Additional file 3 — Regression analysis using generalized estimating equations (GEE) to determine density dependence relative sampling efficiency of the ITT-B and the SRB for An. gambiae s.l. and the Cx. species. Statistical analysis to indicate the sampling efficiency of the ITT-B and the HLC in terms of the vector density. [file 1475-2875-8-197-S3.pdf]

Table S3: Regression analysis using generalized estimating equations (GEE) to determine density dependence relative sampling efficiency of the ITT-B and the SRB for *An. gambiae s.l.* and the *Cx.* species

Species	Alternative/HLC	Versus Alternative method+ HLC [a]		
		Parameter	Estimate [95% CI]	P
An. gambiae s.l.	ITT-B	Log_{10}(HLC+alternative)	NA	0.733[b]
		Intercept	-0.781 [-0.941, -0.621]	<0.001
Cx. species	ITT-B	Log_{10}(HLC+alternative)	NA	0.096[b]
		Intercept	-0.969 [-1.282, -0.650]	<0.001
	SRB	Log_{10}(HLC+alternative)	NA	0.992[b]
		Intercept	-1.960 [-2.399, -1.521]	<0.001

[a] =Reference method

[b] = Not statistically significant and therefore not included in the model

NA=Not applicable

CI=Confidence interval
