# Supplementary material for: Comparative evaluation of the Ifakara tent trap-B, the standardized resting boxes and the human landing catch for sampling malaria vectors and other mosquitoes in urban Dar es Salaam, Tanzania
Source: Malar J. 2009 Aug 12;8:197. doi: 10.1186/1475-2875-8-197 (PMC2734863; doi:10.1186/1475-2875-8-197)
Supplement: Additional file 4 — Abdominal condition scored by the three traps for An. gambiae s.l. and Cx. species and the influence of each trap on the fed mosquitoes determined by binary logistic regression. The data represent statistical analysis of the abdominal status of the three traps. [file 1475-2875-8-197-S4.pdf]

Table S4: Abdominal condition scored by the three traps for *An. gambiae s.l.* and *Cx.* species and the influence of each trap on the fed mosquitoes determined by binary logistic regression

Species	Variable	Fed		
	Trap type	Proportion	Odds [95% CI]	P
An. gambiae s.l.	ITT-B	0.08 [n=135]	1.38 [0.55, 3.42]	0.493
	SRB	0.37 [n=27]	9.53 [3.57, 25.47]	<0.001
	HLC	0.06 [n=143]	1.00[a]	NA
Cx. species	ITT-B	0.06 [n=6661]	6.66 [4.86, 9.12]	<0.001
	SRB	0.21 [n=1351]	29.76 [21.52, 41.15]	<0.001
	HLC	0.01 [n=4975]	1.00[a]	NA

[a]=Reference method

n=Total number of mosquitoes

NA=Not applicable

CI=Confidence interval
