# Supplementary material for: Comparative evaluation of the Ifakara tent trap-B, the standardized resting boxes and the human landing catch for sampling malaria vectors and other mosquitoes in urban Dar es Salaam, Tanzania
Source: Malar J. 2009 Aug 12;8:197. doi: 10.1186/1475-2875-8-197 (PMC2734863; doi:10.1186/1475-2875-8-197)
Supplement: Additional file 5 — Species composition of An. gambiae complex for the ITT-B, HLC and SRB and the influence of each trap upon the proportion of An. gambiae s.s. sampled, as determined by binary logistic regression. The data presented is a summary of statistical analysis of the species composition of the three traps as determined by PCR. [file 1475-2875-8-197-S5.pdf]

Table S5: Species composition of *An. gambiae* complex for the ITT-B, HLC and SRB and the influence of each trap upon the proportion of *An. gambiae s.s*. sampled, as determined by binary logistic regression

Trap type	*An. gambiae s.s*		
	Proportion	Odds [95% CI]	P
ITT-B	0.54 [n=110]	0.52 [0.23, 1.17]	0.115
SRB	0.48 [n=26]	1.80 [0.21, 15.12]	0.588
HLC	0.61 [n=132]	1.00[a]	NA

[a]=Reference trap

n=Total number of mosquitoes

CI=Confidence interval

NA=Not applicable
