# Supplementary material for: Comparative evaluation of the Ifakara tent trap-B, the standardized resting boxes and the human landing catch for sampling malaria vectors and other mosquitoes in urban Dar es Salaam, Tanzania
Source: Malar J. 2009 Aug 12;8:197. doi: 10.1186/1475-2875-8-197 (PMC2734863; doi:10.1186/1475-2875-8-197)
Supplement: Additional file 6 — Comparative evaluation of cost effectiveness of the ITT-B and the HLC for weekly sampling and sampling a single An. gambiae s.l. and Cx. species. A summary of the cost of using the ITT-B and the HLC for sampling An. gambiae s.l. and Cx. species. [file 1475-2875-8-197-S6.pdf]

Table S6: Comparative evaluation of cost effectiveness of the ITT-B and the HLC for weekly sampling and sampling a single *An. gambiae s.l.* and *Cx.* Species

Type of cost	Item	Details	Quantity		Total amount for HLC/ week/ site	Total amount for ITT-B / week/ site
			ITT-B	HLC		
Initial set up costs	Traps	ITT-B	12	NA	NA	$2.10
		Maintained and depreciated over 5 years	NA	7.5 km x $0.50	$4.40	NA
		Bicycles maintenance and depreciation over 5 years	12	NA	NA	$0.37
	Collecting materials	Aspirators and torches	12	12	$0.25	$0.25
Total Initial set up cost per week per sampling site					$4.65	$2.74

Recurring cost	Labour	Adult mosquito catchers	12	12	$5.00	$4.30
		Driver	1	NA	$0.80	NA
		Surveillance coordinators	NA	2	$2.40	NA
	Field work	Fuel and maintenance cost	NA	8km x $0.5	$5.00	NA
	Office materials	Consumables			$1.48	$1.54
	Medical tests				$1.00	NA
	Miscellaneous cost				$2.55	$0.58
Total recurring cost per week per sampling site					$18.23 [a]	$6.42 [a]
Total initial set up and recurring costs per week					$22.88	$9.15
Cost per mosquito caught per week	*An. gambiae s.l.*		0.83	0.73	$24.97[a]	$7.73[a]
	Cx. species		130	67.98	$0.27[a]	$0.05[a]

[a]=Cost of sampling one mosquito calculated by dividing the total recurring cost per week per site by the number of mosquitoes caught per week per sampling site.
